# Dietary Exposure of Nigerians to Mutagens and Estrogen-Like Chemicals

**DOI:** 10.3390/ijerph110808347

**Published:** 2014-08-15

**Authors:** Iyekhoetin Matthew Omoruyi, Derek Ahamioje, Raimo Pohjanvirta

**Affiliations:** 1 Food and Environmental Toxicology Unit, Department of Food and Environmental Hygiene, Faculty of Veterinary Medicine, University of Helsinki, P.O.Box 66, 00014 Helsinki, Finland; E-Mail: raimo.pohjanvirta@helsinki.fi; 2 Department of Basic Sciences, Faculty of Basic and Applied Sciences, Benson Idahosa University, P.M.B. 1100, Benin City, Edo State, Nigeria; E-Mail: dahamioje@biu.edu.ng

**Keywords:** mutagenicity, endocrine-disrupting chemicals, estrogenic activity, processed food, pure water sachet

## Abstract

Food and drinking water are poorly delineated sources of human exposure to chemical food mutagens and endocrine-disrupting chemicals. In this study, we investigated the presence of mutagens and chemicals exhibiting estrogenic activity in the daily diet of Nigerians, using *in vitro* assays. Commercially processed foods or snacks and various brands of pure water sachets were extracted by solid-phase extraction and liquid-liquid extraction, respectively. Mutagenicity was determined by the conventional Ames test and two complementary assays on two strains of *Salmonella* (TA 100 and TA 98), while the estrogenic activity was assessed by a yeast bioluminescent assay, using two recombinant yeast strains (*Saccharomyces cerevisiae* BMAEREluc/ERα and *S*. *cerevisiae* BMA64/luc). A third of the food varieties investigated (chin-chin, hamburger, suya and bean cake) were mutagenic in all three assays, either in the presence or absence of S9 mix. Of the packed water samples, five out of the sixteen investigated (31%), were found to be estrogenic, with estradiol and bisphenol A equivalents ranging from 0.79 to 44.0 ng/L and 124.2 to 1,000.8 ng/L, respectively. Hence, although the current situation in Nigeria does not appear to be substantially worse than, e.g., in Europe, regular monitoring is warranted in the future.

## 1. Introduction

Food and drinking water are major sources of human exposure to both mutagens and endocrine-disrupting chemicals (EDCs) globally [1,2,3,4,5,6,7]. This is alarming in view of the fact that food and water are prerequisites of human life.

The sources of chemical mutagens in food vary remarkably, depending on the foodstuff and processing methodology. However, emphasis has traditionally been placed on reducing the levels of possible mutagenic residues in meat, grain, vegetables *etc.* prior to processing, neglecting the possibility of a less clear-cut risk: the formation of these mutagenic compounds in food as a result of processing. Yet, processed food items are reported to contain chemical substances known to have mutagenic, genotoxic and carcinogenic effects, and thus acting as a key global contributor to human cancer risk [8,9,10,11]. Polycyclic aromatic hydrocarbons (PAHs) and heterocyclic amines have been reported in processed food (mainly meat and fish products) at various concentrations all over the world [12,13,14,15]. The formation of these chemical mutagens during food processing has been demonstrated to depend on a number of factors such as cooking time, method of cooking and type of heat source [3,8,12]. For example, the Ames test shows a correlation between meat-processing temperature and the number of revertants generated per gram of meat [3,4]. Chung *et al.* [12] also reported that charcoal-grilled pork contained higher levels of PAHs (10.2 µg/kg) compared with other methods of processing. Likewise, high concentrations of PAHs have been found in smoked-cured fish in Ghana [13]

The contamination by mutagenic PAHs of thermally treated high-protein foods such as charcoal-grilled meat products is mainly due to the direct pyrolysis of food fats and the deposition of PAHs from smoke produced through incomplete combustion of the thermal agents [16]. Unfortunately, this method of food processing is the method of choice in most developing nations, including Nigeria. Although knowledge of proper processing techniques would help reduce the risk of generating mutagenic compounds in food, a recent study showed that only 4.76% of 63 subjects involved in food processing in Nigeria had a formal training in a food safety/hygiene-related discipline [17]. Similar percentages have also been reported in Kenya and Ghana [18,19].

Regarding EDCs, the bulk of information available is on compounds possessing estrogen-like activity. Phytoestrogens and food contact materials are the main sources of human exposure to xenoestrogens in food [20,21,22]. While the health effects of phytoestrogens remain controversial, synthetic xenoestrogens have been associated with certain cancer types, reproductive disorders, developmental abnormalities and other adverse physiological effects in both humans and wildlife [23,24,25]. In this light, it is quite worrisome that drinking water sources as well as bottled mineral and flavored waters have been reported to contain estrogenic substances [5,6,7,26]. The estrogenic activity in bottled mineral and still water is mainly attributed to the prevailing use of several phthalates and other plasticizers including bisphenol A in packaging materials [5,6]. These chemicals are increasingly raising concern, because they may leach into consumer products in normal use [27,28,29]. There are over 50 chemical compounds authorized for use in food contact materials which are known to have endocrine-disrupting potential [30]. Interestingly, when food contact materials are assessed for their health risk, they are not routinely tested for their endocrine-disrupting potential [31]. However, the Endocrine Society has expressed its concern about the widespread exposure of humans to these chemicals, as they are capable of affecting multiple endpoints within a living system [32].

Chemical mutagens and EDCs in food and water samples have usually been determined by various methods of analytical chemistry. However, these methods suffer from a number of limitations in their ability to elucidate the entire range of chemical mutagens and EDCs in a single experiment, including an unknown number of yet-to-be identified compounds. *In vitro* assays offer the advantage of detecting all substances that contribute to the functional property (mutagenicity or estrogenic activity) being assessed in food, water and environmental samples. Therefore, in the present study, we sought to determine the genotoxic and estrogenic properties of food and water samples by *in vitro* assays. We focused on Nigerian products, because the customary food processing methods there are potentially risky in this regard (see above) and because, to the best of our knowledge, such information does not yet exist in the body of scientific literature.

## 2. Materials and Methods

### 2.1. Materials

All chemicals used in this study were of analytical grade. The NADP and glucose-6-phosphate used were obtained from Roche Biochem (Stockholm, Sweden). Aroclor-induced S9 from rat liver was purchased from Trinova Biochem (Giessen, Germany). Histidine, potassium chloride, magnesium sulfate, potassium phosphate dibasic anhydrous and sodium ammonium phosphate were purchased from Merck AG (Darmstadt, Germany). Magnesium chloride hexahydrate and citric acid monohydrate were acquired from VWR international (Leuven, Belgium). Biotin, tryptophan, methylcellulose (MC), dimethyl sulfoxide (DMSO), benzo[*a*]pyrene, 2-aminoanthracene, sodium azide, estradiol, bisphenol A, progesterone and testosterone were purchased from Sigma-Aldrich (Steinheim, Germany). D-Luciferin was obtained from Biotherma (Handen, Sweden). Yeast nitrogen base medium without amino acids was obtained from Becton Dickinson (Franklin Lakes, NJ, USA).

### 2.2. Microorganisms

The bacteria, *Salmonella enterica* sv. *typhimurium* strains TA 100 and TA 98, were obtained from Pasteur’s Institute (Paris Cedex, France). Two recombinant yeast strains *Saccharomyces cerevisiae* BMAEREluc/ERα and *S*. *cerevisiae* BMA64/luc [33] were used in this study. In the yeast bioluminescent assay, BMAEREluc/ERα served as a reporter strain, in which the ERα is expressed. Upon ligand binding, the dimerized receptor binds the estrogen response elements in the promoter region of the *luc* reporter gene. In *S*. *cerevisiae* BMA64/luc, luciferase is expressed constitutively, and this strain was used for determination of cytotoxicity of the test samples. Both yeast strains are kind gift donations by Dr. Johanna Rajasärkkä of the Department of Food and Environmental Sciences, Faculty of Agriculture and Forestry, University of Helsinki, Finland. Yeasts were grown on Difco Yeast Nitrogen Base medium without amino acids, supplemented with 40% glucose and their respective amino acids.

### 2.3. Cell Line

Human hepatocellular carcinoma-derived cell line (HepG2) was obtained from American Type Culture Collection through LGC standards (Boras, Sweden) and cultured in Eagle’s Minimum Essential Medium (LGC standards) containing 10% heat-inactivated fetal bovine serum (Sigma-Aldrich, Steinheim, Germany). The cells were maintained at 37 °C in a humidified atmosphere of 5% CO_2_ in air atmosphere incubator (NuAire Inc., Plymouth, MA, USA).

### 2.4. Sampling and Sample Preparation

A total of 36 samples (3 lots of 12 varieties) representing commonly consumed, commercially processed food items in Nigeria were evaluated for their mutagenic potential. All varieties were obtained from different vendors since no quality control is carried out in the production of these food items. Moreover, equivalent food products obtained from the same manufacturer have been previously reported to vary in their mutagenic potential [2,4]. Since the major source of xenoestrogens in processed food items are phytoestrogens and food contact materials, and all our food samples were informed to be free of soy (a highly significant source of phytoestrogens) and mostly unpackaged, we targeted water samples as possible sources of exposure to EDCs. Sixteen sachet pure water samples sold in Benin City metropolis, Edo State, Nigeria were acquired for the purpose of this study. Food samples were extracted by solid phase extraction method [4], while possible estrogenic compounds were extracted from the water samples (1000 mL each) by liquid–liquid extraction as described by [34].The final extracts were concentrated to approximately 2 mL using a rotary evaporator, and the concentrates were shipped on ice to the Department of Food Hygiene and Environmental Health, Faculty of Veterinary Medicine, University of Helsinki, Helsinki, Finland. Upon arrival, samples were further concentrated to dryness under nitrogen. Food samples were reconstituted in DMSO, while water samples were reconstituted in 5% ethanol for *in vitro* analyses. Food packaging materials were extracted for possible estrogenic activity as described previously [1].

### 2.5. Cytotoxicity Assays

The cytotoxic effect of the concentrations of food extracts used in this study was investigated by two independent assays measuring trypan blue exclusion and lactate dehydrogenase (LDH) activity as previously described [2]. Briefly, HepG2 cells were grown in 24-well plates (VWR, Finland) for 48 h, and further exposed to different concentrations of food extracts for 4, 24 or 48 h. After exposure, the cells were trypsinized and centrifuged for 5 min at 2500 rpm. Pellets were then resuspended in PBS, after which 10 µL of the cells were mixed with 5 µL (0.8 mM) trypan blue dye for microscopic observation. LDH activity was performed according to the instructions provided in the Cytotoxicity Detection Kit^PLUS^ (LDH), version 6 (Roche Biochem, Stockholm, Sweden).

### 2.6. Mutagenicity Assay

The mutagenic potential of food extracts was initially determined by the standard plate incorporation assay. Samples showing mutagenic potential in this assay were subsequently subjected to “treat-and-wash” as well as methylcellulose overlay assays to ascertain to what degree a localized release of proteins, peptides or histidine from the samples contributed to the outcome.

#### 2.6.1. Standard Plate Incorporation Assay

The standard plate incorporation assay was performed as described by Maron and Ames [35] using *Salmonella* strains TA 100 and TA 98 with and without metabolic activation (S9 mix). The amount of S9 used in the S9 mix was 10%. Water and DMSO were used as negative controls for both strains while sodium azide (0.04 mg/mL) and 2-aminoanthracene (0.02 mg/mL) served as positive controls for TA 100 and TA 98, respectively. Benzo[*a*]pyrene (0.1 mg/mL) was also used as a positive control for both strains. The volume of controls used was 50 µL/plate in triplicate plates. Sodium azide is a known direct mutagen in *Salmonella* TA 100 [36], whereas 2-aminoantracene is metabolically activated by mono-oxygenases of the CYP1A family in rat liver [37]. Likewise, benzo[*a*]pyrene requires metabolic activation for mutagenicity [38].

For all samples, four different concentrations of the food extracts (25, 50, 100 and 200 mg/mL) were tested in triplicate plates (50 µL/plate). The highest concentration (200 mg/mL) was equivalent to 1 g of the food sample. The plates were incubated at 37 °C for 48 h.

The results of the mutagenic activities are presented as the number of revertant colonies per gram of food sample. Only the mean and standard deviation of the highest concentration for all food extracts are shown.

#### 2.6.2. Treat-and-Wash Assay

The treat-and-wash assay was conducted according to the method described by Thompson *et al.* [39]. The protocol applied was as per the standard plate incorporation assay with the exception that the S9 mix, bacteria and sample extract were incubated for 90 min prior to the addition of molten top agar. Briefly, a 500 µL aliquot of S9 mix/phosphate buffer (0.2 M, pH 7.4) was combined with 100 µL each of late-log bacterial culture and sample extract solution in a sterile 15 mL tube. The mixture was incubated for 90 min in a mechanical shaker (180 rpm) at 37 °C. The extended duration of bacterial exposure compensated for the absence of bacterial exposure on plates, as the test sample was washed away prior to plating. After a 90-min preincubation, 10 mL of wash solution (Oxoid No. 2 nutrient broth in phosphate-buffered saline (1:7 v/v)) was added, and the washed bacteria were collected by centrifugation at 2,000 g for 30 min. All but approximately 700 µL of the supernatant was removed and discarded, and the bacteria were resuspended in the residual supernatant prior to plating via top agar.

#### 2.6.3. Methylcellulose Overlay Assay

Methylcellulose overlay assay was performed as previously described [39]. Briefly, a 500 µL aliquot of S9 mix/phosphate buffer (0.2 M, pH 7.4) was combined with 100 µL of late-log bacterial culture in a sterile 15 mL tube. A 2 mL aliquot of the MC overlay suspension was added to the tube, and a 100 µL aliquot of the sample extract solution was added immediately afterward. The mixture was overlaid on a pre-warmed (37 °C) minimal glucose plate. Plates were held at 4 °C for 1 h after plating to ensure gelling of the MC overlay, and subsequently incubated (not inverted) at 37 °C for 48–72 h. The MC overlay was prepared on the day of the test, and the mixture was stirred at 50–60 °C throughout use.

### 2.7. Yeast Bioluminescent Assay

The yeast bioluminescent assay was performed as previously described [1]. Estradiol and bisphenol A were used as positive controls, while progesterone and testosterone served as negative controls.

### 2.8. Statistical Analysis/Interpretation of Data

The mutagenic potency of each food sample was determined from the slope of the linear portion of the dose-response curve by linear regression analysis using the software program Prisma 4.0 (GraphPad software Inc., San Diego, CA, USA). In addition to the requirement of a statistically significant (*p* < 0.05) dose-response effect, only those samples were considered mutagenic whose highest test concentration generated at least twice as many revertants as the negative control (DMSO). For proper interpretation and clarity, the number of revertants obtained was compared with both their experiment-specific controls and aggregate controls across all experiments. The *p* values of these comparisons in the tables are derived from the regression analyses. In the estrogenic activity assays, the fold induction, fold induction corrected (FIC) and limit of detection (LOD) were calculated as described previously [33]. The sigmoidal dose-response curves for increasing concentrations of estradiol and bisphenol A were obtained using Prisma 4.0. The estradiol and bisphenol A equivalents of food samples showing estrogenic activity were calculated from probit transformation of the curves.

## 3. Results

### 3.1. Plate Incorporation Assay: Control Substances

The results obtained with the control substances on both strains of *Salmonella* are presented in Table 1. In the test system, the number of revertants generated by sodium azide was 4–5 fold the negative control (DMSO) in TA 100 strain, both in the presence and absence of metabolic activation (S9 mix). This was an expected outcome, because sodium azide is the recommended direct chemical mutagen for TA 100 strain [36]. Meanwhile, the number of revertants generated by benzo[*a*]pyrene was 3–4 and 2–3 fold that of DMSO with and without metabolic activation, respectively. In *Salmonella* TA 98 strain, 2-Aminoanthracene behaved as expected, with the number of revertant colonies being 17–23-fold higher than that of the control substance, in the presence of S9 mix. On the other hand, only a 2–3-fold increment was observed in the absence of S9 mix. No mutagenic effect/potency was observed with benzo[*a*]pyrene in the absence of metabolic activation; co-incubation with S9 resulted in an approximately three-fold increment in colony formation.

**Table 1:** Ranges for revertant colonies obtained with control substances in the standard plate incorporation assay.

Controls	Number of Revertant Colonies			
	Respective Controls *		Aggregate Controls **	
	+S9	−S9	+S9	−S9
***Salmonella* TA 100**				
Water	134.3–177.7	112.7–134.3	161.8 ± 22.4	125.2 ± 11.0
DMSO	138.7–166.3	111.0–128.3	155.3 ± 17.9	120.2 ± 11.3
Sodium azide	568.7–651.0	432.7–516.3	621.6 ± 78.8	470.1 ± 42.7
Benzo[*a*]pyrene	438.0–517.3	248.0–270.0	471.6 ± 48.5	260.7 ± 75.7
***Salmonella* TA 98**				
Water	32.3–42.7	19.0–34.0	37.4 ± 3.1	25.0 ± 4.8
DMSO	35.7–36.7	19.0–24.0	36.2 ± 2.8	21.5 ± 3.6
2-Aminoanthracene	624.3–837.7	55.0–69.3	754.2 ± 68.2	61.4 ± 11.2
Benzo[*a*]pyrene	102.0–122.0	20.3–29.7	116.8 ± 10.4	24.0 ± 5.1

Notes: * Range; ** Mean ± SD.

### 3.2. Plate Incorporation Assay: Test Substances/Food Samples

The mutagenic activity of commercially processed food items, obtained by the standard plate incorporation assay, is presented in Table 2 and Table 3. The majority of samples investigated (75%) exhibited fairly high mutagenic activity (the maximal responses being comparable to those elicited by benzo[*a*]pyrene), mainly in *Salmonella* TA100 strain. However, there was notable lot-to-lot variation.

In TA 100 strain, chin-chin, hamburger, suya and bean cake were the most mutagenic food samples investigated. The number of revertants generated by these samples was over twice that of DMSO in all the batches analyzed and mostly independent of the S9 mix. A somewhat surprising result was found with the potato products (french fries and potato chips), as at least one of the lots of both products proved directly mutagenic. Roasted maize, plantain chips and coconut-candy did not show any evidence of mutagenic potency in this strain (Table 2).

In *Salmonella* TA 98, only three food or snack varieties (potato chips, peanut and suya) exhibited mutagenic potency in at least one of the batches investigated (Table 3). Suya displayed the most coherent outcome with all its three batches being mutagenic in the presence of S9 mix. In support of the result with TA 100, the same batch of potato chips (number 3) exhibited direct mutagenicity also in TA 98.

**Table 2:** Number of revertants generated by the highest concentrations (1.0 per g of food sample) of food extracts on *Salmonella* TA 100 (mean ± SD) in the standard plate incorporation assay.

Food Products	Revertants per Gram					
	Batch 1		Batch 2		Batch 3	
	+S9	−S9	+S9	−S9	+S9	−S9
Doughnut	319.0 ± 12.1 ^†,ф^	254.0 ± 14.0 ^†,ф^	201.3 ± 07.1	198.7 ± 10.0	209.7 ± 14.0	186.0 ± 13.1
Chin-chin	285.7 ± 15.5 ^†^	191.3 ± 10.3	460.3 ± 28.3 ^†,ф^	330.7 ± 75.8 ^†,ф^	469.3 ± 71.9 ^†,ф^	257.7 ± 26.1 ^†,ф^
Hamburger	353.3 ± 43.5 ^†,ф^	248.3 ± 79.2 ^†,ф^	469.3 ± 44.4 ^†,ф^	350.0 ± 45.8 ^†,ф^	212.3 ± 38.0	302.7 ± 72.9 ^†,ф^
Coconut-candy	265.7 ± 12.4	198.0 ± 06.9	245.7 ± 18.9	203.0 ± 09.7	180.0 ± 28.9	137.7 ± 05.8
French fries	255.0 ± 47.8	304.3 ± 33.8 ^†,ф^	208.0 ± 26.9	157.7 ± 12.3	189.3 ± 19.6	308.3 ± 43.0 ^†,ф^
Potato chips	159.0 ± 10.8	159.3 ± 04.6	210.7 ± 15.1	193.3 ± 07.0	188.7 ± 06.5	256.7 ± 11.4 ^†,ф^
Plantain chips	124.7 ± 11.8	109.3 ± 01.5	159.3 ± 27.3	192.7 ± 11.1	185.7 ± 11.1	154.7 ± 06.5
Peanut	293.3 ± 30.6 ^†^	242.7 ± 04.6 ^†,ф^	339.0 ± 62.6 ^†,ф^	272.7 ± 61.6 ^†,ф^	215.0 ± 26.2	175.3 ± 28.4
Roasted maize	252.3 ± 23.0	187.3 ± 04.7	176.7 ± 29.9	154.0 ± 07.0	215.7 ± 20.0	202.7 ± 18.5
Suya	383.0 ± 20.7 ^†,ф^	240.7 ± 10.0 ^†,ф^	401.7 ± 12.1 ^†,ф^	296.0 ± 06.0 ^†,ф^	308.7 ± 14.6	263.3 ± 05.8 ^†,ф^
Fried chicken	138.7 ± 06.0	133.0 ± 08.5	394.7 ± 14.7 ^†,ф^	204.3 ± 06.0	381.0 ± 35.5 ^†,ф^	156.0 ± 23.5
Bean cake	312.0 ± 25.4 ^†,ф^	241.7 ± 36.8 ^†,ф^	365.0 ± 22.6 ^†,ф^	159.0 ± 25.9	294.7 ± 12.3	278.7 ± 09.1 ^†,ф^

Notes: **^†^**: Significantly different from respective controls (*p* < 0.05); **^ф^**: Significantly different from aggregate control (*p* < 0.05).

**Table 3:** Number of revertants generated by the highest concentrations (1.0 per g of food sample) of food extracts on *Salmonella* TA 98 (mean ± SD) in the standard plate incorporation assay.

Food Products	Revertants per Gram					
	Batch 1		Batch 2		Batch 3	
	+S9	−S9	+S9	−S9	+S9	−S9
Doughnut	40.3 ± 8.4	22.0 ± 8.2	30.7 ± 11.6	19.0 ± 4.6	28.0 ± 3.0	19.0 ± 6.1
Chin-chin	34.0 ± 1.7	23.3 ± 2.1	31.0 ± 7.9	20.0 ± 5.3	37.7 ± 6.5	27.3 ± 4.2
Hamburger	46.0 ± 14.0	26.3 ± 3.1	31.7 ± 4.5	20.7 ± 3.5	43.0 ± 7.0	20.7 ± 2.1
Coconut-candy	38.7 ± 4.9	24.3 ± 1.6	40.7 ± 6.1	25.3 ± 5.1	34.7 ± 9.3	21.3 ± 1.5
French fries	32.3 ± 1.5	36.0 ± 6.6	30.3 ± 3.5	17.0 ± 2.0	31.3 ± 4.0	18.7 ± 6.4
Potato chips	36.3 ± 5.9	26.7 ± 3.8	33.0 ± 4.6	26.3 ± 4.0	31.7 ± 2.1	72.7 ± 18.0 ^†,ф^
Plantain chips	29.0 ± 5.6	29.0 ± 2.6	32.3 ± 2.9	18.7 ± 5.5	38.3 ± 6.1	21.0 ± 4.6
Peanut	69.7 ± 5.6	34.7 ± 4.0	78.0 ± 12.5 ^†,ф^	37.0 ± 14.9	35.0 ± 2.0	27.3 ± 5.0
Roasted maize	24.3 ± 2.1	20.0 ± 2.0	31.7 ± 4.9	28.0 ± 5.3	27.3 ± 5.0	23.7 ± 7.4
Suya	86.7 ± 5.8 ^†,ф^	27.7 ± 2.5	83.0 ± 3.5 ^†,ф^	19.0 ± 4.6	97.3 ± 7.6 ^†,ф^	20.7 ± 3.5
Fried chicken	32.0 ± 5.0	28.3 ± 5.1	24.0 ± 4.0	19.7 ± 3.8	28.3 ± 3.2	20.7 ± 2.5
Bean cake	32.0 ± 5.3	11.0 ± 1.7	28.7 ± 8.5	22.3 ± 5.8	32.3 ± 3.1	21.0 ± 2.0

Notes: **^†^**: Significantly different from respective controls (*p* < 0.05); **^ф^**: Significantly different from aggregate control (*p* < 0.05).

### 3.3. Modified Ames Tests

To ascertain to which degree a localized release of proteins, peptides or histidine contributed to the mutagenicity test results obtained with the standard plate incorporation assay, “treat-and-wash” as well as MC overlay assays were performed (Table 4 and Table 5). The outcome proved to depend on bacterial strain, type of food, S9 status, and assay. For some food extracts initially found to be mutagenic in the standard plate incorporation assay, the number of revertants decreased below the two-fold limit level in comparison with the negative control. Hence, the original Ames test result was in these cases interpreted to be of secondary nature and not due to genuine mutations. However, in a large number of cases, the food extracts were mutagenic in all three assays both in the presence and absence of S9 mix. For some food items (hamburger, suya and bean cake), a single lot was mutagenic in all three assays but only in the presence of S9 mix. In the absence of S9, the outcome with these three products varied. In contrast to this pattern, a single batch of hamburger (batch 2) was mutagenic in all three assays, both in the presence and absence of S9 mix. A couple of surprises also emerged in these complementary assays. Extracts of fried chicken (batch 2) and bean cake (batch 2) that required metabolic activation for their mutagenicity in the standard plate incorporation assay, were, unexpectedly, directly mutagenic in the treat-and-wash assay in *Salmonella* TA 100 strain (Table 4). One of these samples (bean cake, batch 2) behaved the same way also in the MC overlay assay (Table 4). An identical shift from indirect to direct mutagen was recorded in *Salmonella* TA 98 strain for extracts of peanut (batch 2) and suya (batch 2) (Table 5).

### 3.4. Cytotoxicity Assays

The cytotoxicity of the four concentrations of all food extracts was determined by both trypan blue exclusion and LDH secretion assays in HepG2 human hepatocellular carcinoma cells. The non-survival percentage of HepG2 cells in the trypan blue exclusion test did not exceed 50%. Also, there was a significant difference between the positive control (lysis solution) and the test samples in the amount of LDH released. Hence, the extracts were classified non-cytotoxic in these assays following exposure for 4, 24 or 48 h.

### 3.5. Estrogenic Activity Assay: Control Substances

**Figure 1:**
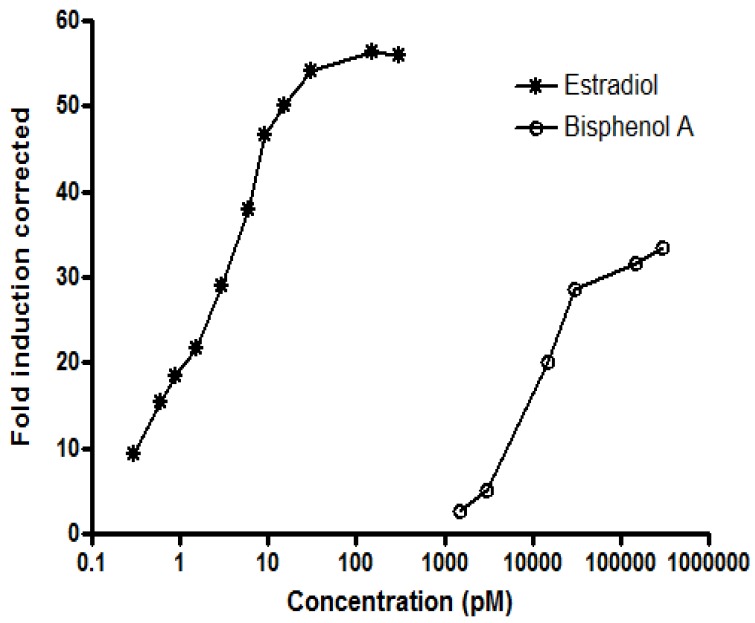
Dose-response curves of increasing concentrations of estradiol and bisphenol A.

The positive and negative control compounds used in this study behaved as expected with the *S. cerevisiae* BMAEREluc/ERα yeast strain. Both positive controls (estradiol and bisphenol A) produced a sigmoidal dose-response curve (Figure 1), while the negative controls (progesterone and testosterone) did not elicit any luciferase activity in the test system. This is in keeping with previously published data [29,40]. The limit of detection (LOD) in the yeast bioluminescent assay was 2.4-fold induction corrected (FIC), corresponding to 76 fM and 1.2 nM of estradiol and bisphenol A, respectively.

**Table 4:** Number of revertants in the treat-and-wash as well as methylcellulose overlay assays generated by the highest concentrations (1.0 per g of food sample) of food extracts showing mutagenic potential on *Salmonella* TA 100 (mean ± SD) in the standard plate incorporation assay.

Food Product	Batch	Revertants per Gram			
		Treat-and-Wash Assay		Methylcellulose Overlay Assay	
		+S9	−S9	+S9	−S9
Doughnut	1	115.0 ± 4.2	84.3 ± 8.7	215.0 ± 34.6	191.3 ± 10.6
Chin-chin	1	192.7 ± 17.5	58.7 ± 7.8	128.3 ± 9.8	95.0 ± 11.4
Hamburger	1	633.0 ± 23.3 *	124.7 ± 10.6	330.0 ± 10.4 *	166.7 ± 28.3
French fries	1	121.3 ± 11.7	103.0 ± 1.4	84.3 ± 22.6	86.0 ± 4.9
Peanut	1	108.0 ± 7.1	123.5 ± 13.4	182.3 ± 26.2	93.0 ± 15.6
Suya	1	366.0 ± 22.6 *	113.0 ± 1.4	382.0 ± 17.2 *	271.7 ± 9.4 *
Bean cake	1	736.3 ± 85.1 *	156.0 ± 13.9 *	401.0 ± 28.4 *	238.2 ± 12.1
Chin-chin	2	618.7 ± 58.7 *	32.3 ± 12.5	126.0 ± 8.5	125.7 ± 10.6
Hamburger	2	397.7 ± 21.2 *	408.0 ± 32.5 *	304.7 ± 19.4 *	263.3 ± 9.3 *
Peanut	2	141.3 ± 23.3	42.0 ± 11.3	344.3 ± 31.8 *	201.3 ± 2.1
Suya	2	181.0 ± 16.9	240.0 ± 42.4 *	165.0 ± 10.6	76.0 ± 5.0
Fried chicken	2	174.3 ± 12.4	150.3 ± 12.8 *	192.3 ± 13.9	138.3 ± 12.4
Bean cake	2	470.0 ± 16.3 *	260.7 ± 33.2 *	324.7 ± 28.6 *	271.3 ± 19.4 *
Chin-chin	3	126.3 ± 12.0	131.7 ± 10.6	183.3 ± 9.9	164.0 ± 16.3
Hamburger	3	135.0 ± 18.4	126.3 ± 9.2	166.7 ± 0.7	162.0 ± 22.6
French fries	3	139.7 ± 6.8	97.0 ± 8.5	110.0 ± 3.5	95.3 ± 2.1
Potato chips	3	194.7 ± 11.6	108.3 ± 9.4	90.7 ± 10.0	106.0 ± 18.2
Suya	3	179.0 ± 9.9	209.0 ± 19.7 *	194.3 ± 7.8	271.0 ± 14.4 *
Bean cake	3	371.3 ± 12.1 *	228.0 ± 23.3 *	267.7 ± 14.3	290.0 ± 21.9 *
Fried chicken	3	117.7 ± 12.3	108.3 ± 11.4	158.0 ± 12.8	124.3 ± 11.4

Note: *: Significantly different from control (*p* < 0.05).

**Table 5:** Number of revertants in the treat-and-wash as well as methylcellulose overlay assays generated by the highest concentrations (1.0 per g of food sample) of food extracts showing mutagenic potential on *Salmonella* TA 98 (mean ± SD) in the standard plate incorporation assay.

Food Product	Batch	Revertants per Gram			
		Treat-and-Wash Assay		Methylcellulose Overlay Assay	
		+S9	−S9	+S9	−S9
Suya	1	78.0 ± 8.5 *	17.0 ± 1.4	48.0 ± 4.8	24.3 ± 2.7
Peanut	2	30.0 ± 8.5	158.0 ± 51.0 *	31.3 ± 5.0	84.3 ± 9.6 *
Suya	2	33.3 ± 5.0	154.0 ± 0.0 *	29.3 ± 1.9	78.7 ± 6.6 *
Potato chips	3	48.3 ± 7.2	21.7 ± 4.1	33.0 ± 2.4	19.3 ± 2.1
Suya	3	42.0 ± 9.9	18.0 ± 3.1	39.7 ± 5.2	26.7 ± 3.8

Note: *: Significantly different from control (*p* < 0.05).

### 3.6. Estrogenic Activity of Pure Water Sachets

The estrogenic activities of the 16 pure water samples investigated ranged from below LOD to 44.0 ng/L (median: 23.0 ng/L) estradiol equivalent (the amount of estradiol needed to bring about the same effect as the sample analyzed in an assay specific for estrogens) concentrations (EEQs). Five out of the 16 sachets produced luciferase activities greater than the LOD. The positive water samples were coded W1 to W5 (Table 6). W1 had the lowest value of 0.79 ng/L or 124.2 ng/L estradiol *vs.* bisphenol A equivalent concentrations, respectively. Concurrently, the highest values found (for sample W2) extended to 44.0 ng/L (estradiol equivalent) or 1000.8 ng/L (bisphenol A equivalent).

**Table 6:** Estradiol (EEQ) and bisphenol A (BPAEQ) equivalent concentrations of sachet water samples.

Sample Code	Water Samples		Sachet/Packaging Material	
	EEQs (ng/L)	BPAEQs (ng/L)	EEQs (pg/L)	BPAEQ (pg/L)
W1	0.79	124.2	14.5	224.0
W2	44.0	1000.8	<LOD	<LOD
W3	28.0	597.8	10.2	186.1
W4	23.0	442.8	<LOD	<LOD
W5	15.0	269.7	<LOD	<LOD
Median	23.0	443.0	12.4	205.0
Average	7.0	152.0	2.0	26.0

As an attempt to further trace the origins of the estrogenic activities observed, the sachets themselves were analyzed for possible leaching of estrogenic substances into the water. The packaging material of three out of the five positive samples did not generate any positive signal in the yeast-based assay. However, a feeble response was obtained from the packaging material of the two other samples (Table 6).

## 4. Discussion and Conclusions

Processed food items and bottled water are consumed in increasing quantities all over the world. Therefore, it is of utmost importance to ensure that in addition to their microbial safety, the products do not contain chemicals which might pose a toxicological risk to consumer health. A conceivable potential risk in this regard is the formation of genotoxic compounds during the processing of foodstuffs and leaching of food contact materials into food and water. Regular screening studies are necessary to verify that the methods used by food vendors are appropriate and sound also from this point of view. The present investigation aimed at exploring the current situation in Nigeria.

To the best of our knowledge, this is the first study on mutagenicity of food products from Africa. To compare our data with those of previous studies is challenging because of the paucity of published data on mixture effects combined with the wide variation in food types in different parts of the world. However, it is possible to compare foodstuffs based on the number of revertants their extracts generate in the Ames test and its derivatives, and we will utilize this approach.

Food processing methods as well as the sales of processed food items in Nigeria are poorly—if ever—regulated. Furthermore, it has been reported that the majority of Nigerians involved in food processing do not have formal training on food safety issues or related techniques [17,41]. This may bear on the present finding that the majority of food items (75%) investigated were mutagenic in the standard plate incorporation assay for at least one of the three batches when studied in *Salmonella* TA 100 strain. On the other hand, in *Salmonella* TA 98 strain only 25% of food extracts were found to yield a mutagenic response, possibly due to a weaker sensitivity of this strain compared with TA 100 or to the type of mutagens present.

The conventional Ames test outcome cannot, however, be taken at its face value in the case of food extracts as these may be sources of localized release of proteins, peptides or histidine itself onto the bacterial plates [2]. To prevent this potential misinterpretation of ostensible mutagenicity, “treat-and-wash” as well as methylcellulose overlay assays were performed for all samples eliciting a positive outcome in the conventional Ames test. The results of these complementary assays were consistent for some samples (bean cake, suya, hamburger, fried chicken and chin-chin), further reinforcing our initial findings with the Ames test. Extracts of bean cake and suya stood out from among the positive samples. All batches of bean cake exhibited mutagenic activity in the treat-and-wash assay with the *Salmonella* TA 100 strain, both in the presence and absence of S9 mix. One of these lots (batch 1) generated a conspicuously high number of revertants, almost five-fold its control (DMSO), with metabolic activation. A similar situation was observed with the MC overlay assay, as all bean cake samples were mutagenic in *Salmonella* TA 100 strain in the presence of S9 mix. Similarly, all batches of suya were consistently mutagenic in the treat-and-wash assay, with the *Salmonella* TA 100 strain, either in the presence or absence of S9 mix.

Bean cake is commonly consumed in different parts of Nigeria, irrespective of ethnicity, religion or social status. A probable explanation for the mutagenicity test results observed with extracts of bean cake is in the method of its processing. Bean cake is processed by deep-frying for several minutes. Deep-frying has previously been reported to result in the formation of mutagenic and genotoxic compounds in the final product [42]. Food vendors in Nigeria are also known to repeatedly reuse their frying oil, which is often already of questionable quality, for several days or weeks. This may have contributed to the high number of revertants obtained with extracts of bean cake and fried chicken. Double heat-treatment of cooking oil has been shown to cause an increase in the genotoxic activity of food products [43,44]. During frying, cooking oil undergoes deterioration through various chemical and physical processes such as oxidation, polymerization, hydrolysis and cyclization, leading to the formation of both volatile and non-volatile undesirable by-products [43]. These derivatives are partially absorbed by the fried food, which thus becomes carcinogenic [45]. For example, the PAH compounds benzo[*a*]pyrene and benzo[*a*]anthracene are all well-known human carcinogens which have been detected in different types of cooking oil [45].

The positive mutagenicity test results obtained with suya were not unexpected. Suya is 100% beef, and it is a special type of delicacy, mainly consumed in Nigeria, irrespective of social status. All suya products are processed the same way: by charcoal-grilling. After processing, the products are left to be heated on the charcoal for several hours, until they are purchased. This processing method typically explains the reason for the mutagenicity test results obtained with extracts of suya in our study. The contamination of thermally treated high-protein foods, such as charcoal-grilled meat products, by PAHs and heterocyclic aromatic amines is well established [12,46,47,48]. The building up of PAHs in this case is due to their generation by direct pyrolysis of food fats and the direct deposition of PAHs from smoke produced through incomplete combustion of the thermal agent [16]. Heterocyclic aromatic amines, in turn, are formed through the condensation of creatine/creatinine and the strecker degradation radicals (pyridines and pyrazines) generated from the reaction of sugars and amino acids during the Maillard reaction [49]. The present findings are worrisome, because meat-cooking habits have been linked with several forms of cancer [50,51,52,53]. In Argentina, for example, cooking meat at a high temperature and close to the cooking source has been linked with increased incidence of colorectal cancer [54]. This is also the case in Hawaii and the Netherlands [55,56]. More recently, a number of PAHs have been reported in different types of smoked meat in Serbia, Latvia and Sweden [14,57,58]. However, no nexus has been established in relation to increased incidence of cancer in these countries. In Nigeria, there is a paucity of data on the incidence of different cancer types, but the two major forms, breast and prostate cancers, may be increasing [59]. Both of them have been associated with meat-cooking habits [60].

Hamburger products have previously been reported as a major source of chemical food mutagens to consumers [38,61]. The results obtained in our study further reinforce this view, as two different lots of hamburgers examined were found to be mutagenic in all three assays in *Salmonella* TA 100 strain, with one of the lots (batch 2) being both directly and indirectly mutagenic in all three assays. Stavric *et al.* [61] previously reported hamburger products purchased in Ontario, Canada, to be mutagenic in a similar assay, but only with *Salmonella* TA 98 strain. The number of revertants generated in that study ranged from 63 to 1042 rev/g (average: 199 rev/g). This is in contrast to our study, in which hamburger products were only mutagenic with the *Salmonella* TA 100 strain, and not TA 98. In a recent study in Finland [2], the number of revertants generated with extracts of hamburger products were slightly lower than those obtained in this study, both with *Salmonella* TA 100 and 98 strains. Although these findings might seem to implicate the current cooking methods of hamburgers in Nigeria, the present outcome may not be entirely attributable to the processing methods. This is due to the fact that high levels of potassium bromate, a well-known mutagen and human carcinogen, have been detected in bread in different parts of Nigeria [62,63,64]. In one of these cases, Alli *et al.* [62] found that even the lowest level of potassium bromate in their bread samples was over 150 times higher than the maximal permissible limit.

Overall, the mutagenicity test outcome of our study is in keeping with previously published data on food mutagenicity elsewhere [3,4,61], but somewhat at odds with a recent study published in Finland, where only 40% of the processed food items investigated showed mutagenic properties in the conventional Ames test [2]. In further contrast with the current findings, for most food varieties in the study by Omoruyi and Pohjanvirta [2], only a single batch proved positive. This may reflect more refined food processing techniques in Finland as compared with Nigeria.

In Nigeria, it is estimated that about 25% and 53% of people living in urban and rural areas, respectively, lack access to pure, portable water [65]. This is related to recent outbreaks of several water-borne diseases in major states of the country, specifically cholera [66,67]. It has prompted entrepreneurs to continuously establish water plants, in which pure water samples are mainly packaged in plastic sachets.

Our study demonstrates that pure water sachets may contain estrogen-like chemicals. Five of the 16 samples investigated were discovered to be estrogenic in our *in vitro* test system, with EEQs ranging from 0.79–44.0 ng/L. Both the frequency of positive samples and their concentrations were actually lower than we feared, considering that the proprietors of pure water sachet factories in Nigeria are principally entrepreneurs with little or no knowledge of water quality (microbiological, physicochemical or toxicological). There are two recent studies carried out in Europe in which estrogenic activity of water samples was assessed by a comparable *in vitro* yeast assay to that of ours. Pinto and Reali [68] analyzed mineral waters packed in polyethylene terephthalate (PET) bottles in Italy. The levels they detected varied from 0.03 through 23.1 ng/L (mode 9.5 ng/L) EEQs. Somewhat surprisingly, tap water made of either surface water or ground water contained approximately 15 ng/L EEQs. In another study, Wagner and Oehlmann [6] determined estrogenic activities in 20 major brands of bottled water in Germany. Twelve of these samples proved positive with the levels ranging from 2.64 to 75.2 ng EEQ/L (average 18.0 ng/L). Interestingly, in their material, the highest estrogenic activities were recorded for waters packaged in either non-reusable PET or reusable glass bottles, and even water packed in Tetra Pak™ bricks contained levels that were similar to those found in our study (14–44 ng/L). Thus, substances exhibiting estrogen-like activity are common in water samples in both industrialized and developing countries.

It is widely believed that the decline in male reproductive functions, increased incidence of different cancer types amongst young men and women and neurobehavioural diseases observed in the population of different countries may, at least partly, be attributable to exposure to estrogenic compounds, particularly during the intrauterine phase or during critical periods of postnatal development [23,24,25,69]. Studies in recent years have shown, for example, that the commonly used plasticizer, di(2-ethylexyl)phthalate (DEHP), alters gene expression in rats and that, at appropriate concentrations, it alters the development of the central nervous system in the fetus [70]. Similarly, certain compounds, such as benzophenone used as food contact material, are reported to almost completely block the 17β-hydroxysteroid dehydrogenase type 3 enzymes that are required for testosterone synthesis [71].

The presence in or leaching into water samples of endocrine-disrupting chemicals is influenced by a number of factors such as storage conditions, exposure to sunlight and ambient temperature [72,73]. Unfortunately, the environmental conditions in Nigeria (abundant sunlight and high temperature) tend to favor the migration of endocrine-disrupting chemicals from the packaging materials into water, as, for example, during transport of water containers. Therefore, sachets of water stored or transported in less appropriate conditions than our samples would be at risk of containing higher concentrations of estrogenic substances.

The bulk of dietary xenoestrogen exposure for adults has been proposed to emanate from dairy products, and total daily intake of estrogens has been estimated to be 80–100 ng [74]. Assuming an average daily water consumption of 3 L at Nigerian latitude [75], in the worst-case scenario based on our sample material, the intake from pure water sachets would double the estimated exposure. Hence, every effort should be taken to reduce the estrogen levels in these waters in the future.

In conclusion, the results obtained in our study show that both commercially processed food items and sachet-packed pure water sold in Nigeria, are sources of mutagen and estrogen-like chemicals, respectively. Although their concentrations are not alarming in the light of food and water analyses from other countries, measures should be taken to reduce them further and monitor their levels regularly. Since the number of samples examined here was relatively low, further survey studies are also warranted.
